# Using corrected Cone-Beam CT image for accelerated partial breast irradiation treatment dose verification: the preliminary experience

**DOI:** 10.1186/1748-717X-8-214

**Published:** 2013-09-13

**Authors:** Jiazhou Wang, Weigang Hu, Gang Cai, Jiayuan Peng, Ziqiang Pan, Xiaomao Guo, Jiayi Chen

**Affiliations:** 1 Department of Radiation Oncology, Fudan University Shanghai Cancer Center, Shanghai, China; 2 Department of Oncology, Shanghai Medical College, Fudan University, 270 DongAn Road, Shanghai 200032, China

**Keywords:** CBCT, APBI, Image correction

## Abstract

**Background:**

Accurate target localization is mandatory in the accelerated partial breast irradiation (APBI) delivery. Dosimetric verification for positional error will further guarantee the accuracy of treatment delivery. The purpose of this study is to evaluate the clinical feasibility of a cone beam computer tomographic (CBCT) image correction method in APBI.

**Methods:**

A CBCT image correction method was developed. First, rigid image registration was proceeded for CTs and CBCTs; second, these images were separated into four parts; then, ratio images for each of the four parts of planning CTs/CBCTs were calculated and filtered to reduce the high spatial frequency; finally, the enhanced CBCT images were generated combing the four parts. An anthropomorphic thorax rando phantom was used to evaluate the feasibility and accuracy of the CBCT correction method. The CBCT images of consecutive 10 patients receiving APBI were corrected using the above method and dosimetric variations were evaluated. Each set of CBCT is composed of three images: one acquired after skin-marker setup, one after online setup correction and one after treatment delivery.

**Results:**

The phantom study showed the improved accuracy of dose calculation with corrected CBCT. The Dose Volume Histogram (DVH) difference between the planning CT and corrected CBCT is less than the difference between the planning CT and original CBCT. The maximum dose difference between the corrected CBCT and planning CT is 0.8% in PTV_EVAL V_100_, which is 3.8% between original CBCT and planning. In the patient study, 67.4% of fractions benefit from CBCT setup corrections in PTV_EVAL D95, while in 47.4% of the fractions, reduced dose coverage was found on the post-treatment CBCT. Overall, the CBCT based initial setup correction guaranteed target dose coverage in 9 patients.

**Conclusions:**

A generic CBCT image correction algorithm was created and proved to be easily implemented in clinic. Compared to the original CBCT, the corrected CBCT has more accuracy in dose calculation. The CBCT guided APBI based on initial skin setup is not sufficient to guarantee the accurate dose delivery throughout each fraction. The long treatment delivery time may compromise the target coverage benefits in some patients.

## Background

Radiation therapy plays an important role in patients with early breast cancers receiving breast conservative treatment (BCT) [1]. Compared to the whole breast irradiation (WBI), the accelerated partial breast irradiation (APBI) has better normal tissues sparing and has proved its safety in selected low risk patients [2]. Unlike the WBI, the APBI has larger fractional dose and smaller target volume. Therefore, it is more sensitive to setup errors.

CBCT images for each treatment fraction can provide sufficient anatomical information for correcting setup errors [3]. Besides that, breast shape variation may also compromise the accuracy of partial breast planning [4]. Therefore, it is crucial to find out the extent of dosimetric change following these variations and displacements.

The use of CBCT images for verifying the actual delivered dose has attracted increasing concerns. Numbers of studies have investigated the utilization of CBCT for dose calculation, such as the direct method with established specific CBCT Hounsfield Unit (HU)-density calibration curve [5-7]; the planning CT-based method with correcting the CBCT using planning CT information [8]; and the projection scatter correction method which reduces the scatter before CBCT image reconstruction [9-11]. Among them, the HU-density calibration method, which uses phantom or specific population to get the HU-density curve without complex image process algorithm can be easily to implemented in clinic [12,13].However, in certain cases, severe image scatter artifact will cause dose calculation error [7,14,15]. The planning CT-based correction and projection scatter correction require complex image processing algorithm. Both of them state the fact that it is critical to find a simple CBCT correction method for fractional APBI dose verification.

The purpose of this study is to evaluate the feasibility and dosimetric accuracy of a simple planning CT-based CBCT correction method for APBI dose verification. Additionally, the preliminary clinical results in our department of performing CBCT based dose verification were reported.

## Methods

### CBCT acquisition and correction workflow

All the CBCT images used in this study were acquired on the Elekta Synergy X-Ray Volume Imaging (XVI) system (Elekta Synergy S, Elekta Oncology Systems, Crawley, UK). The details of patient position and CBCT imaging protocols have been reported in our previous study [16]. Briefly, all patients were fixed on the breast board and at a 200° arc (instead of 360°). CBCT imaging was performed for avoiding the gantry-couch collision. Approximately 360 projections were collected over 200° rotation of the gantry (range from 250° to 90° for the left breast tumors and from 180° to 30° for the right breast tumors). The scanning parameters were S20 collimator, 120 kV, 36.1 mA-s. No bow-tie filter was used in this study. CBCT images were reconstructed with 1 mm cubic voxels and averaged in the longitudinal direction for 3 mm slice thickness. Planning CT was acquired with a Brilliance Big Bore CT scanner (Philips Medical Systems, Cleveland, OH) with 5 mm thickness and 0.8–1.0 mm pixel size.

A planning CT-based correction was performed to reduce the scatter effect and complete the CBCT with full anatomy. The CBCT is corrected using the following method:

1) To rigidly register the planning CT and CBCT images and resample the CBCT to planning CT’s image resolution.

2) To separate the planning CT and CBCT into 4 different parts as air, bone, lung, and soft tissue and generate four masks for each part.

3) To individually calculate the local ratio images for the 4 parts using the equation (1):

(1) Ratio_image=planningCT/CBCT

4) To filter the 4 ratio images to reduce the high spatial frequency information.

5) To generate the enhanced CBCT image for each part by multiplying the filtered ratio image with the corresponding CBCT image.

6) To use the corresponding mask to filter the CBCT image background and keep only the corresponding part of the image. Combine the 4 filtered enhanced CBCT images to get the corrected CBCT image.

7) The limited CBCT field of view (FOV) is then completed using the planning CT.

**Figure 1:**
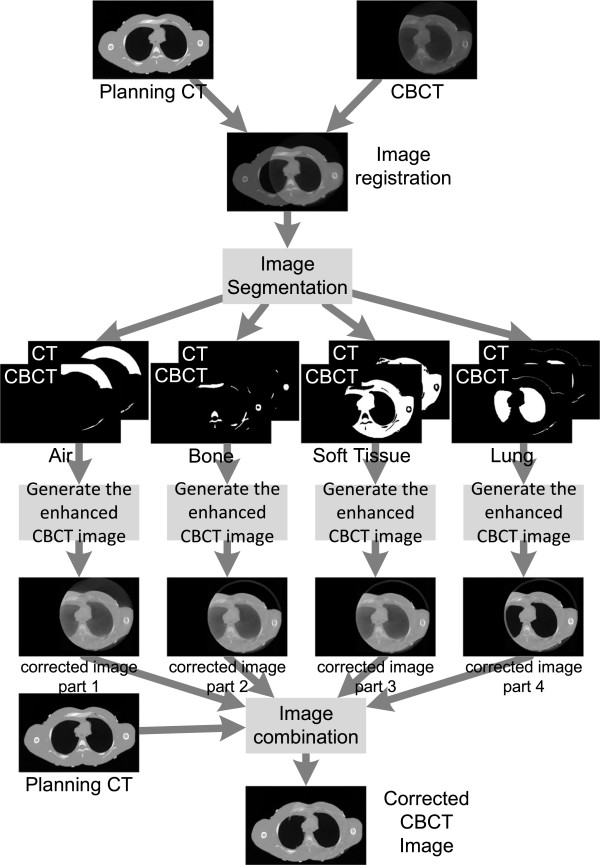
The diagram of the correction method of the CBCT correction algorithm.

Figure 1 shows the overall procedure of the correction.

### Rando phantom validation

The phantom validation of the CBCT correction algorithm was performed on an anthropomorphic thorax phantom (ChenDu Dosimetric Phantom, named by ICRU Report No.48, 1992). The corrected CBCT image was generated with the aforementioned method. A hypothetical target and sensitive structures were contoured by one attending radiation oncologist. A planning target volume (PTV) mimicking the actual treatment was delineated in the left breast in the planning CT. A PTV_EVAL is defined from a copy of the PTV minus tissue that is within 5 mm from the breast surface and posterior to the breast tissue. The sensitive structures include ipsilateral lung and heart.

The planning CT, original CBCT and corrected CBCT were imported to the Pinnacle treatment planning (TPS, Philips Medical Systems, v8.0 m, Milpitas, CA). All contours were copied to the original CBCT and corrected CBCT after rigid registrations. An APBI plan was generated in the planning CT in the Pinnacle system as Baglan et al. described [17]. This method was actually applied for all the patients receiving APBI.

The plan was copied to the original and corrected CBCTs for dose calculations. The original CBCT based dose calculation was performed using HU-density calibration method [5]. The dose distributions and dose volume histograms (DVHs) generated using the planning CTs, corrected CBCTs and the original CBCTs were compared.

### Clinical implementation

Ten APBI patients were enrolled in this study (7 left-sided and 3 right-sided). Institutional based ethical committee approved all patients’ treatments. The target volumes were delineated according to Radiation Therapy Oncology Group (RTOG) 0413 Phase III protocol [18]. Plans using non-coplanar 6MV beam arrangement as stated previously were generated and manually optimized to meet the clinical requirements. In order to improve the dose conformity, an additional anterior electron beam was used in one patient.

The dose prescription was 38.5 Gy delivered in 10 fractions, with a total duration of 5-7 days. All treatment plans had that more than 95% of PTV EVAL was completely encompassed by the 95% isodose line (D95 ≥ 36.6Gy), while maintaining a minimum dose greater than 93% (Dmin ≥ 35.8Gy) and a maximum dose less than 110%(Dmax ≤ 42.4Gy).

The treatment was delivered twice daily with an interfractional interval of at least 6 hours. At the first fraction on each treatment day, 3 CBCT images were scanned and named as CBCT_marker_ (obtained after skin-marker setup), CBCT_pre_ (obtained after online setup correction and prior to the treatment), and CBCT_post_ (obtained after treatment delivery).

All corrected CBCT images were transferred to the TPS for dose calculation. The PTV_EVAL was modified by the oncologist based on the corrected CBCT so as to keep it 5 mm beneath the skin. The DVH was calculated for the PTV_EVAL, ipsilateral lung and heart (if the tumor is located in left breast). The minimum dose, maximum dose and D_95_ to PTV_EVAL were obtained from these DVHs. The ipsilateral breast V_50_, V_100_, ipsilateral lung V_30_, heart dose V_5_ (tumor locates at the left side) were recorded. The dose distribution between the planning CT and all 3 CBCT images were compared. A paired sample test was performed to test the variations. The conventional significance level of p <0.05 was adopted.

## Results

### Rando phantom validation

**Figure 2:**
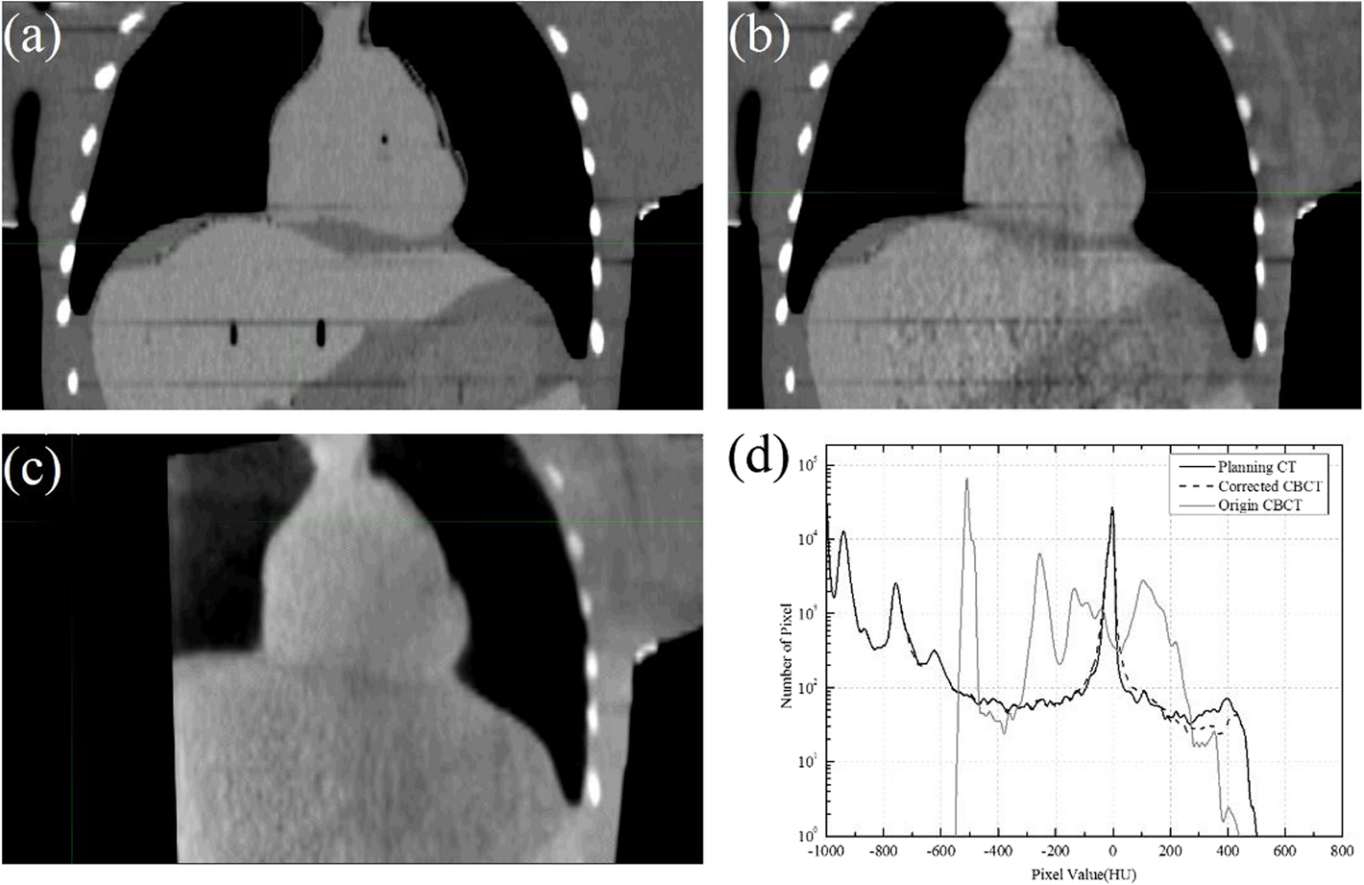
**Slice of the rando phantom image and image histogram. (a)** A coronal plane of planning CT. **(b)** Corresponding plane of the corrected CBCT, **(c)** Corresponding plane of the CBCT. **(d)** Image histogram of the planning CT (dark solid line), corrected CBCT (dash line) and original CBCT (grey solid line).

Figure 2a-c show the same slice of the planning CT, corrected CBCT and original CBCT images of the rando phantom, respectively. Figure 2d shows the image pixel histogram of the planning CT, corrected CBCT and original CBCT. Apparently, the distribution of pixel intensity of corrected CBCT is closer to the reference CT than the original CBCT. The detailed dosimetric data are listed in Table 1. The maximum percentage difference between the corrected CBCT and planning CT is 0.8% in PTV_EVAL V_100,_ which is 3.8% between original CT and planning CT. Compared to the original CBCT, the corrected CBCT has more accuracy in dose calculation.

**Table 1:** Dosimetric data from the corrected and original CBCT in reference to the planning CT

	**Planning CT**	**Corrected CBCT**	**Original CBCT**	**Percentage difference (planning CT vs. corrected CBCT)**	**Percentage difference (planning CT vs. original CBCT)**
PTV_EVAL V_100_	96.6%	95.8%	92.9%	-0.8%	-3.8%
PTV_EVAL V_90_	100.0%	100.0%	100.0%	0.0%	0.0%
PTV_EVAL D_95_ (Gy)	38.6	38.6	38.3	0.0%	-0.8%
Ipsilateral lung V_30_	2.0%	2.0%	2.0%	0.0%	0.0%
Heart V_5_ (Gy)	0.0	0.0	0.0	0.0%	0.0%

### Patients study

Three CBCT_marker_ and 13 CBCT_treated_ were not acquired or missing. Altogether, a total of 134 CBCT images were collected for patient study. The rigid registration metrics were obtained from actual treatments and then used for generating the corrected CBCT images. Three corrected CBCT images named as CoCBCT_marker_, CoCBCT_pre_, CoCBCT_post_ were created corresponding to the skin markers, pre-treatment correction and post-treatment correction, respectively.

**Figure 3:**
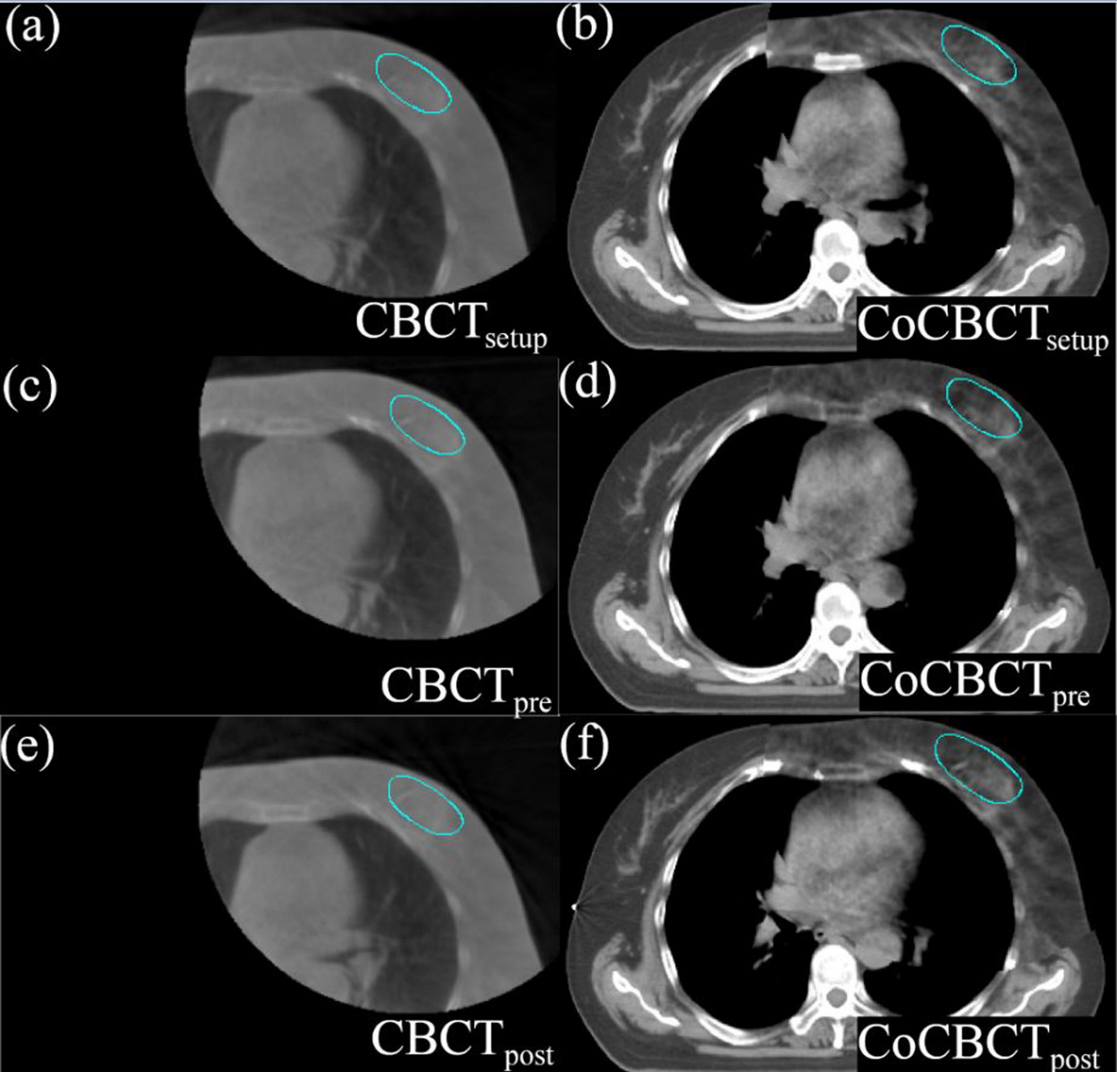
**The axial views of original CBCT images and corrected CBCT images for one fraction of patient2. (a)** CBCT after skin-marker setup **(b)** corrected CBCT after skin-marker setup **(c)** CBCT after online setup correction **(d)** corrected CBCT after online setup correction **(e)** CBCT after treatment delivery **(f)** corrected CBCT after treatment delivery.

Figure 3a-f shows an example of the three CBCT images and their corresponding corrected CBCT images for one fraction (patient2). Similar to the phantom study, the correction algorithm shows good CBCT correction results with improved image quality on the actual patient.

The whole dosimetric data were obtained from all the corrected CBCT images for evaluation. Table 2 shows the comparison of dose to the PTV_EVAL, ipsilateral breast, ipsilateral lung and heart for the whole CBCT group.

**Table 2:** Actual dose delivery obtained from three CoCBCTs in reference to the planning CT

		**Planning CT**	**CoCBCT**_**marker**_	**CoCBCT**_**pre**_	**CoCBCT**_**post**_
PTV_EVAL	D_95_	38.1 ± 0.3	37.6 ± 1.3	38.1 ± 0.7	38.0 ± 1.0
	Dmin	35.1 ± 5.1	27.8 ± 8.1	31.7 ± 5.1	31.8 ± 4.1
	Dmax	40.0 ± 5.9	41.3 ± 0.7	41.2 ± 1.1	39.9 ± 6.7
Breast V_100_ (%)		18.3 ± 5.4	17.0 ± 4.5	17.2 ±3.9	17.4 ± 4.6
Breast V_50_ (%)		49.0 ± 12.1	44.1 ± 10.5	46.0 ± 10.5	47.4 ± 10.9
Ipsilateral lung V_30_ (%)		4.9 ± 2.2	4.2 ± 3.0	4.3 ± 1.9	4.2 ± 3.1
Heart V_5_ (%)		25.7 ± 30.7	26.1 ± 26.4	25.8 ± 25.2	21.0 ± 20.0

Noticeably, the dosimetric parameters of PTV_EVAL D_95_ and Dmax meet the protocol criteria for all the three CBCT images. However, compared to the planning dose, V90 and Dmin of the PTV_EVAL were lower in the CBCT_marker_, while similar to those in the CBCT_pre_- or CBCT_post_. For the normal tissue doses, the actual doses from all 3 CBCT data sets were lower than the planning CT. These indicate that there still exist some underdose in the target in actual deliveries should initial CBCT with skin setup were not performed. Normal tissues sparing were sufficient with current treatment protocol.

**Figure 4:**
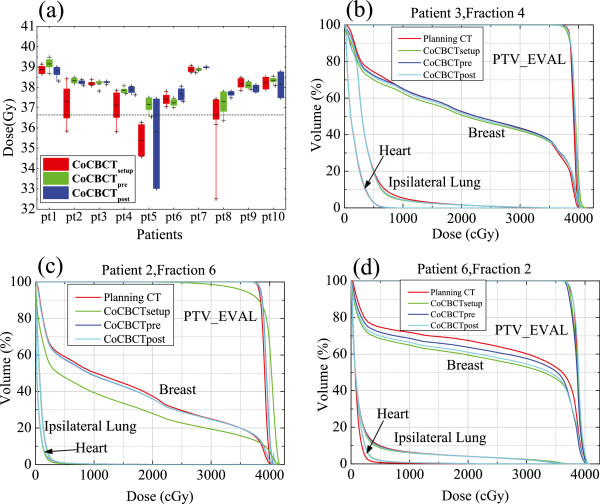
**CBCT based DVH. (a)** The D95 to the PTV_EVAL for the whole patient group. **(b-d)** Three DVHs of patients.

Figure 4a shows the statistical results of D_95_ for the 10 patients. Initial skin marker based setup insures enough doses for 6 patients. CBCT based setup correction helps to improve the target coverage in almost all patients. In four patients (pt2, pt4, pt5, and pt8), significant coverage improvement with CBCT_marker_ was achieved_._ The dosimetric benefit of CBCT_marker_ was maintained in 8 patients; however, such benefit is lost for 2 patients verified with CBCT_post_ (pt5 and pt10). Statistically, for the PTV_EVAL D95, 67.4% of fractions benefited from initial CBCT based setup correction, while in 47.4% of fractions reduced dose coverage was found in the post-treatment CBCT verifications. Our study shows that the CBCT based setup correction guarantees target dose coverage in 9/10 patients. Three representative DVHs were plotted in Figure 4b-d. These DVHs are in good agreement for all three CBCTs (Figure 4b. pt3, fraction4), significant benefit from CBCTs (Figure 4c, pt2, fraction6) and slight benefit from CBCTs (Figure 4d, pt6, fraction2). Again, these DVHs display the dosimetric benefits for CBCT based setup corrections.

## Discussion

In this study, a simple CBCT image correction method was created and implemented for the APBI. The Rando phantom study shows the clinical accuracy and feasibility of using the correction method for fractional dose verification of APBI. Our clinical result shows that the corrected CBCT based calculation is a practical tool to verify the actual delivery doses.

Although the specific CBCT HU-density table can be used to directly calculate the dose calculation, it is not feasible for APBI cases with limited FOV. First, the HU stability in CBCT is highly phantom size dependent [19]. The different patient size will lead to apparent HU varietiation, which may result in error on dose calculation. Second, when breast board is used, it is quite complicated to have a 360° CBCT scanning. A 200° scanning CBCT image has only partial anatomy structures, which cannot be used for dose calculation directly [20]. Compared to the full arc scanning, the CBCT image quality is degraded with 200-degree scanning. Therefore, a simple planning CT-based post-processing method must be implemented for completing the anatomical structure and improving the image quality for accurate dose calculation.

For APBI, the major concerned parts for dose calculation are breast (soft tissue), rib, lung and air. Thus, 4-part segmentation was performed in our algorithm. After enhancing and combining the four parts of CBCT image, a corrected CBCT image was completed with planning CT. This method is based on the correction algorithm from Marchant [21], while additional lung segmentation was used and its application was extended from prostate to breast.

When the FOV of the image is smaller than the patient body, the partial object effect was significant for some patients. Segmentation error may occur at the edge of the image in this study. However, as no beam would go through those areas and they are also remote from the target volume, the influence was negligible. Furthermore, such effect can be avoided with more sophisticated segmentation methods in the future. Many of the algorithm parameters, such as the thresholds in segmentation, are empirically chosen in this study. These parameters are adequate for cases of APBI, and they can be further optimized when applying to other sites.

The phantom study validates the accuracy of using the correction method for dose calculation. After phantom validation, this method was implemented in the clinical scenario to verify the dosimetric change before and in the course of single fraction of APBI. According to the 134 CBCT data sets from 10 patients, it is evident that the CBCT guided setup correction improved the target coverage compared to skin-market setup and maintained the normal tissue sparing. However, it was found that in 47% of fractions, there existed target coverage reduction in the end of single fraction after verifying with the post treatment CBCT images. More over one patient (pt5) had PTV_EVAL D95 less than 95%, indicating a target underdose. This phenomenon implies that the benefit of pre-treatment CBCT correction may not be maintained throughout the course of one fraction of APBI. Two major reasons may explain. One is the intrafractional error. Generally, the treatment delivery time is longer than a conventional one. In this study, the mean treatment time for the breast APBI was 25-30 minutes including the setup, CBCT imaging and treatment delivery. The mean beam delivery time for all the fields was 10-15 min. Therefore, the intrafractional error may be important in some patients who are less tolerable for relative long immobilization time. Patient 5 is an example who had axillary dissection instead of sentinel node biopsy, which led to limited upper arm mobility when the APBI started. Another reason for the difference may be caused by the contour error. Although one attending radiation oncologist delineated all contours on the pre- and post- CBCT images for reducing the inconsistent, there might still exists some inevitable differences, which may also explain the lower Dmin in the delivery.

## Conclusions

A generic CBCT image correction algorithm was created. The method has sufficient accuracy for CBCT based dose verification and can be easily implemented in the fractionated APBI patients. The CBCT guided APBI based on initial skin setup is not sufficient to guarantee the accurate dose delivery throughout each fraction. The long treatment delivery time may compromise the target coverage benefits in some patients.

## Competing interests

This research is supported by grants from the National Nature Science Foundation of China (81172504) and the Natural Science Foundation of Shanghai, China (10ZR1406700).

## Authors’ contributions

GC, ZP, XG, JC carried out patient treatment. JW, WH, JP carried out the study. JW, WH, JC conceived of the study, and draft the manuscript. All authors read and approved the final manuscript.
